# Integrated Metabolomic, Molecular, and Morphological Insights into the Degradation of Polychlorinated Biphenyls (PCB) by *Priestia megaterium* MAPB-27

**DOI:** 10.1021/acsomega.5c07925

**Published:** 2025-10-27

**Authors:** Monika Sandhu, Atish T. Paul, Prabhat N. Jha

**Affiliations:** † Department of Biological Sciences, 29794 Birla Institute of Technology and Science Pilani, Pilani Campus, Pilani 333031, Rajasthan, India; ‡ Department of Pharmacy, 29794 Birla Institute of Technology and Science Pilani, Pilani Campus, Pilani 333031, Rajasthan, India

## Abstract

Polychlorinated biphenyls (PCBs) are persistent organic pollutants that cause profound deleterious effects on the environment and human health. Exposure to PCBs and biphenyl can induce changes in cellular metabolite levels. However, metabolic responses to utilize and adapt to PCBs are not well understood. Therefore, this study meticulously examined the PCB degradation potential, gene expression, and metabolic responses of *Priestia megaterium* MAPB-27 exposed to biphenyl. MAPB-27 showed growth and chemotaxis toward PCB degradation intermediates such as biphenyl, dihydroxy biphenyl, benzoate, and catechol. We employed GC-MS/MS to elucidate disparities in the main metabolic pathways in the biphenyl-exposed MAPB-27 through variations in metabolite composition and PCB biodegradation, while Field-emission scanning electron microscopy (FESEM) was used to study cell morphology. GC-MS/MS analysis highlighted the degradation of trichlorobiphenyl, tetrachlorobiphenyl, pentachlorobiphenyl, and hexachlorobiphenyl by *P. megaterium* MAPB-27, exhibiting 92.5, 62.9, 3.7, and 2.4%, respectively. GC-MS/MS analysis identified 4-dihydroxy-2-oxo-valerate, benzoic acid, and 2,3-dihydroxybenzoic acid as the major degradative metabolites in MAPB-27. MAPB-27 extract also contains metabolites with a wide range of direct industrial applications, such as poly­(3-hydroxybutyrate) (3-hydroxybutyrate), a biobased organic acid (3-hydroxypropionoic acid), and antibacterial and antifungal compounds (phenyllactic acid, 4-hydroxyphenyllactic acid, and β-sitosterol). Glyoxylate and dicarboxylate metabolism and fatty acid biosynthesis were observed to be the active metabolisms in MAPB-27 grown in biphenyl-supplemented Minimal Medium. Overall, the results of this study provided important insights into microbial adaptation to biphenyl and the biodegradation of PCB. Thus, the *P. megaterium* MAPB-27 strain can be used for the development of efficient PCB biodegradation strategies and for the exploration of industrial applications.

## Graphical abstract

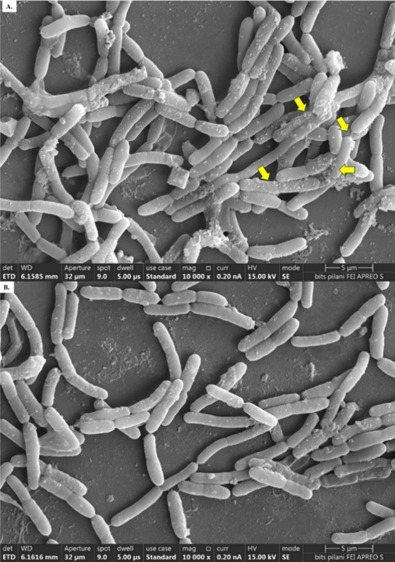

## Introduction

Polychlorinated biphenyls (PCBs) are persistent organic pollutants (POPs) that consist of a biphenyl scaffold with a chlorine functionality. Depending on the number and position of chlorine atoms in the ring, there are 209 different congeners and 10 different homologues of PCBs.^1^ They are employed extensively in industry because of their stable chemical and physical characteristics. However, PCBs are classified as POPs with high toxicity, and they adversely affect the environment and human health. Therefore, an effective approach to remediate soil and water from PCBs takes an hour.

For the breakdown of persistent organic pollutants such as PCBs, bioremediation is a possible substitute for traditional techniques, including physical and chemical procedures. Utilizing the bacteria’s capacity to use organic pollutants as their only source of carbon is the main goal of bioremediation. Mineralization, in which an organic pollutant serves as the only carbon source, and cometabolism, in which an inducer is needed as the second carbon source and simultaneously transforms the target pollutant, are the two methods of biodegradation. *Acinetobacter*, *Pseudomonas,* and *Rhodococcus* bacterial strains have been shown to effectively degrade PCBs when inducers are used.^2^ Biphenyl, the fundamental scaffold of all PCB congeners, is the most often employed inducer to enhance PCB biodegradation.^3,4^ According to reports, biphenyl causes the ring cleavage hydroxylating dioxygenase (ARHD) enzyme, which starts the PCB degradation process. Genes for aromatic ring cleavage have been employed as indicators to determine the catabolic capacities of bacteria and have offered a fascinating perspective on the role the bacteria play in reacting to PCB contamination. The major component of ARHD is the step that limits the rate of aromatic hydrocarbon biodegradation.^5^ By breaking down the contaminant with several enzymes engaged in biodegradation pathways, the native bacteria flourish in the contaminated area. The upper and lower biodegradation processes are part of the entire biphenyl/PCB catabolic pathway.^6^ Initially, the PCB degrader cleaves the two-, three-carbon bond by biphenyl-2,3-dioxygenase to compound II cis-2,3-dihydro-2,3-dihydroxy biphenyl, leading to ring opening and generating chlorobenzoic acid (CBA) and 2-hydroxy-penta-2,4-dienoic acid. Further, cis-2,3-dihydro-2,3-dihydroxy biphenyl converted into 2,3-dihydroxy biphenyl (III), 2-hydroxy-6-oxo-6-phenylhexa-2,4-dienoate (IV), 2-hydroxy-penta-2,4-dienoate (V), and 4-dihydro-2-oxo-valerate (VI).^7^ Benzoic acid and chlorobenzoic acid (CBA) have been identified as dead-end intermediates during biphenyl degradation.^8^ However, benzoic acid and CBA could be further converted or oxidized by bacteria that have benzoate catabolic enzymes.^9^ Therefore, it is crucial to identify the metabolites formed in the biodegradation process and the adaptive mechanisms to counteract the stress. Bacteria have different adaptation mechanisms to combat fluctuating environmental conditions, such as limited nutrient or oxygen availability. Depending upon the types of carbon sources available and in varying abundance, regulatory responses may prioritize one compound over the rest, resulting in a distribution of metabolite fluxes.

Bacilli are considered potential bioremediation agents capable of degrading several toxic substances.^10,11^ There are several reports on members of the genus *Bacillus* degrading various xenobiotic compounds such as polyaromatic hydrocarbons (PAH), dyes, pesticides, and the remediation of heavy metals.^12–14^ Furthermore, strains from the genus *Bacillus* isolated from contaminated soil utilize organic pollutants as their sole source of carbon and energy, suggesting their active adaptive mechanism against environmental stress. Thus, Bacillus spp. could fulfill a vital role in the degradation of organic pollutants in the environment. However, very scarce information exists on the overall adaptive responses and the metabolites exhibited by bacteria exposed to organic pollutants.

Although several species of *Bacillus* can degrade various xenobiotic compounds, the PCB biodegradation potential of Bacilli is relatively rare. In our previous study, *Prestia megaterium* MAPB-27 was reported to be isolated from PCB-contaminated nearby soil of the Bhilai Steel plant, Chhattisgarh, India. *Prestia megaterium* MAPB-27 exhibited the potential to degrade biphenyl^15^ and hence selected for further studies. This study aims to highlight its PCB biodegradation ability and metabolic response and propose novel uses for MAPB-27 in bioremediation research on its broader capabilities and traits. The main metabolic fingerprints in biphenyl breakdown and adaptation mechanisms were identified by using metabolomics based on gas chromatography–mass spectrometry (GC-MS/MS). This is the first research on the biodegradation potential of *P. megaterium* on PCB to counteract stress and the metabolomics profile based on GC-MS/MS.

## Materials and Methods

### Preparation of Bacterial Culture for PCB Biodegradation Ability

#### Bacterial Culture and Growth Conditions of MAPB-27

To investigate the MAPB-27 morphology and metabolomics involved in biphenyl degradation, the MAPB-27 strain was grown to the late log phase (OD600 = 0.8) in an incubator shaker (Metrex Scientific, New Delhi) at 30 °C. After being aseptically moved to a 15 mL Falcon tube, the suspension was centrifuged for 10 min at 8000 × *g*. Following collection, the cell pellet underwent two rounds of washing in a 1× Minimal Medium (MM). The 5× composition of MM (g/L) includes disodium phosphate (33.9), potassium phosphate (15.0), sodium chloride (2.5), ammonium chloride (5.0), with a pH of 6.8 ± 2. To serve as the inoculum, the pellet was reconstituted in 1 mL of MM. In 30 mL screw-capped vials, 4.5 mL of MM enriched with biphenyl (200 mg L^–1^) was mixed with 0.5 mL of cell suspension and shaken at 30 °C and 150 rpm for 72 h. Following incubation, the isolate’s metabolomic profile and FESEM image were analyzed by growing MAPB-27 in biphenyl MM media. As a control, the isolate was cultivated in a 0.2% glucose MM.^15,16^

### Study of the Cellular Morphology of MAPB-27 Using FESEM

To investigate how biphenyl affected the cell shape, isolated MAPB-27 was subjected to an FESEM examination. To assess the cell shape, the bacterial cells were cultured in LB and MM supplemented with biphenyl 200 mg L^–1^. Before adding biphenyl to MM, MAPB-27 was cultivated in 0.2% glucose to achieve the threshold density. Once growth was achieved, cells were harvested and washed twice to remove glucose. Further, it was used as an inoculum in biphenyl-supplemented media. After that, they were incubated at 30 °C for 72 h. Following the incubation time, the smear was formed on a glass slide and fixed for one h using a 2.5% (v/v) glutaraldehyde solution. The fixed bacterial cell was gradually dried with ethanol at intervals of 10 min, resulting in ethanol dehydration of 50–100% (v/v). The immobilized bacterial cells were coated with gold sputter for 30 s. After that, the bacterial cells were examined at various magnification levels using a 20 kV FESEM (FEITM Thermo Fischer Scientific, Apreo, USA).^16^

### Chemotaxis Assays toward the Metabolites Produced during Biphenyl Biodegradation

In order to facilitate the biodegradation of pollutants found in soil, research has shown that bacteria migrate toward chemoattractants like PCB.^17^ Modified swarm and drop tests were used to investigate the MAPB-27’s chemotaxis.^18^ Before placing the plates into the swarm plate medium (MM with 0.16% Bacto agar), biphenyl (final concentration of 0.2 mM) was added for the swarm plate assay. After carefully pouring 100 μL of the stimulated and cleaned cell suspension (OD600 2.0) in MM into the center of the plate, the sample was incubated at 30 °C.

MM consisted of 0.30% agar and 1 mM PCB intermediates, such as biphenyl, dihydroxy biphenyl (DHB), benzoate, and catechol, as the only carbon sources for the drop assay. At 30 °C, MAPB-27 was cultured in LB until it reached the log phase (OD600 = 0.4). After that, the bacterial cells were centrifuged at 8000 × *g* for 10 min. The pellet was washed twice with MM and suspended in the drop assay medium. It was then poured into Petri plates. Chemotactic rings were assessed for PCB intermediates, i.e., biphenyl, DHB, benzoic acid, and catechol, by placing the crystals of intermediates (1 mM) at the center of the plates. Glucose was used as a positive control. The plates were incubated for 24 h at 30 °C. The turbid rings observed around the center after incubation indicate the positive chemotactic movement of the bacteria toward the chemoattractant compound.

### PCB Congener Biodegradation Assay Media

For the biodegradation of PCB congener by MAPB-27, 10% (*v/v*) inoculum was used. 4.5 mL of MM enriched with PCB congeners mix (Sigma-Aldrich, Germany) comprising tri-, tetra-, penta-, and hexachlorinated biphenyl (1 mg/mL) was mixed with 0.5 mL of the resultant cell suspension. The monoculture was incubated at 30 °C with shaking at 150 rpm. MM supplemented with PCB congeners without an inoculum was used as a control. After incubating for 30 days, the contents were extracted to analyze the residual PCBs.^16^

### PCB Congener Extraction and GC-MS/MS Analysis

After the incubation period, an internal standard solution of decachlorobiphenyl (0.10 mg/L) was added to the medium. PCB congeners were extracted with a mixture of *n*-hexane and acetone (1:1; *v/v*) and placed in an ultrasonic bath at 30 °C and 15 Hz for 20 min to release the residual PCBs from the bacterial cell. The solvent was collected and dehydrated by filtration via anhydrous Na_2_SO_4_. The solvent was evaporated using a rotary evaporator, and the concentrated extract was dissolved in isooctane. Furthermore, using previously optimized GC settings^16^ with the SH RTX-5Sil column (30 m × 0.32 mm × 0.25 μm), GC-MS/MS was used to assess the biodegradation of the treated and control sample extracts. The areas of specific peaks in the chromatogram of the samples were measured and compared to those of control samples supplemented with the PCB congener without inoculum. The residual PCB congener was estimated by plotting the calibration curve of PCBs at different concentrations of the standard solution (mg/L) with trichlorobiphenyl (*y* = 2284.9*x* – 25298; *R* ^2^ = 0.9842), tetrachlorobiphenyl (*y* = 826.14*x* – 9516; *R* ^2^ = 0.9847), pentachlorobiphenyl (*y* = 939.79*x* – 9499.5; *R* ^2^ = 0.9864), hexachlorobiphenyl (*y* = 788.97*x* – 7596; *R* ^2^ = 0.9842), and decachlorobiphenyl (*y* = 597.06*x* – 6731.5; *R* ^2^ = 0.9867). The recoveries of PCB congeners were in the range of 96.98–99.10%, while the recovery of decachlorobiphenyl from the standards was in the range of 97.12–98.1%.

### GC-MS/MS Metabolomic Study of MAPB-27

#### Extraction and Derivatization of Metabolites Accumulated in MM Enriched with Biphenyl

To determine the metabolites produced during biphenyl breakdown, the media were extracted after incubation. The media were extracted three times using ethyl acetate after they were acidified with HCl (6N) to reach a pH of 2. A rotary evaporator was used to concentrate the extract.

Trimethylchlorosilane (TMCS) (99:1, v/v) and 100 μL of bis­(trimethylsilyl) trifluoroacetamide (BSTFA) were used to derivatize the extracts for 60 min at 37 °C and 15 min at 60 °C, respectively.^19^

#### GC-MS/MS Identification of the Metabolites Produced in Biphenyl-Supplemented MM

The RXi-5SilMS fused silica column was utilized to separate the metabolites produced in biphenyl-supplemented MM. The column temperature was programmed from 50 °C (2 min hold) to 80 °C at 10 °C/min (5 min hold), 100 °C rise at 5 °C/min (1 min hold), and finally to 300 °C at 15 °C/min (10 min hold) with 1 mL/min of column flow rate. The temperatures of the injection and detector were kept at 280 and 300 °C, respectively. MS was run in full scan mode from *m*/*z* 45 to 500 at a scan rate of 1.68 scans s^–1^ for the identification of compounds by mass spectral NIST14 library.^15,16^

### PCR Amplification of ARHD

To confirm the presence of the gene encoding aromatic ring hydroxylating dioxygenase in the MAPB-27, *the ARHD* gene was amplified using specific primers based on gene information obtained from NCBI. The sequence of forward and reverse primers was 5′-GACCAGCTGGAGAAGCAGAT-3′ and 5′-TGAACCCCTTCGACAGATTC-3′, respectively. The PCR reaction was performed to amplify the ARHD gene in MAPB-27 using specific primers based on gene information obtained from NCBI. PCR amplification was carried out in a reaction volume of 25 μL containing 2.5 μL of Taq DNA polymerase buffer (10×), 0.35 μL of Taq DNA polymerase (3U), 0.3 μL of dNTP (2.5 mM each), 1.0 μL of primer (10 mM each), and 3 μL (50 ng) of the template. Thermocycler T100 (BioRad, Germany) was set for an initial denaturation step of 4 min at 95 °C, followed by 25 cycles of denaturation at 94 °C for 30 s, annealing at the respective temperature for each pair of primers for 45 s (Table 2), extension at 72 °C for 30 s, and a final extension step at 72 °C for 5 min. The PCR amplicon was analyzed on a 1.5% agarose gel under a UV gel documentation unit (Biorad, USA). The amplicon was purified and sequenced using the dideoxy chain terminator method in the DNA sequencing facility of AgriGenome Pvt. Ltd., Kochi. Amplified sequences were compared with the National Centre for Biotechnology Information (NCBI) public database using the BLAST tool (http://www.ncbi.nlm.nih.gov/BLAST/).

### RNA Extraction and Reverse Transcription Polymerase Chain Reaction (RT qPCR)

qPCR was used to examine the gene expression of ARHD, which is dependent on the time and biphenyl concentration. The bacterium MAPB-27 was cultivated in MM enriched with biphenyl at varying doses (100–300 mg L^–1^). The cultures of bacteria were taken at 72 and 120 h into the incubation period. Following incubation, the bacterial culture was centrifuged for 8 min at 10,000 × *g* at 4 °C to pellet it. Following the modified Trizol procedure,^20^ total RNA was extracted and kept at −80 °C. 1% agarose gel electrophoresis was used to confirm the integrity of the RNA. The Nanodrop spectrophotometer (Simplinano, USA) was used to determine the concentration and purity of the RNA. The cDNA was made in order to serve as a reverse transcription template by using Verso cDNA Synthesis kit (ThermoScientific, USA). A 20 mL portion of the reaction mix containing 1 mL of Verso enzyme mix, 2 mL of dNTP mix, 1 mL of RNA primer, 1 mL of RT enhancer, 4 mL of 5× cDNA synthesis buffer, 1 mg of template RNA, and RNase-free water was used for reverse transcription. The reaction mixture was incubated at 42 °C for 30 min, and then the enzyme was inactivated at 95 °C for 2 s. cDNA was stored at −20 °C for further gene expression studies.

### qPCR Analysis for the Expression of ARHD

For PCR and RT-qPCR experiments, a set of 16S rRNA primers and genus-specific primers that target the degradation pathway’s ARHD was the same. DNA (2 mL) from the experimental group and the control group was used as a template for RT-qPCR. The reaction mixture contained 2× Power SYBR Green Master mix (Applied Biosystems TM), 1 mL of forward and reverse primer, 1 mL of cDNA template, and nuclease-free water to make up to 10 mL. The cycling conditions consisted of an initial step of 5 min at 95 °C, followed by 40 cycles of denaturation at 94 °C for 30 s, annealing at 57 °C for 45 s, and elongation at 72 °C for 5 min. RT-qPCR was used to assess the melting curve and efficacy of the chosen primer set using a CFX96 real-time detection system (BioRad, USA). Based on the 2 RT-qPCR triplicates of each biological replicate, the fold change was used to calculate the gene expression level^21^ in ARHD over the 16S rRNA abundance.

### Metabolite Set Enrichment Analysis

Metabolite set enrichment analysis was performed to identify the active metabolism in the biphenyl-grown MAPB-27 cell. The metabolic pathways were mapped using MetaboAnalyst 5.0, an online program that analyzes metabolites.^22^ The functional interpretation of metabolomics data sets was done using the over-representation analysis (ORA) pathway method. The Kyoto Encyclopedia of Genes and Genomes (KEGG) Pathway serves as a reference database for metabolic pathway enrichment. A list of differentially abundant metabolites was used as input for ORA.^23,24^ After the identified metabolites were converted into a KEGG identifier, they were used as input to MetaboAnalyst 5.0. The metabolic pathways in the treatment and control groups were mapped for enrichment analysis using well-annotated KEGG ID molecules (i.e., those in pathway libraries and metabolite sets).

### Statistical Analysis

Every experiment was conducted three times, and the results are shown as ± standard error mean. The one-tailed test was performed for metabolite set enrichment analysis, and a *p*-value below 5% is considered a statistically significant result. One-way analysis of variance was used to compare the biodegradation data (ANOVA). Mean, SD, or ns *P* > 0.05 or ^∗^ *P* < 0.05 or ^∗∗^ *P* < 0.01 as compared were used to summarize the data.

## Results

### FESEM Analysis Revealed Reduced Cell Size, EPS Production, and Vesicle Formation by MAPB-27 in the Presence of Biphenyl

FESEM is an advanced microscope technique with high magnification and the ability to observe minute features of the bacterial cell. Thus, the impact of biphenyl on the surface and cellular morphology of the control and treated MAPB-27 cells was examined using FESEM-based imaging. Figure 1 displays an enlarged picture of the bacterial isolate MAPB-27 cultured in LB and MM supplemented with biphenyl at 10,000× magnification. The average cell size of MAPB-27 was found to be 4.975 ± 0.02 and 4.758 ± 0.01 μm when grown in control and biphenyl-treated conditions, respectively. The treated cells produced membrane vesicle-like structures on the MAPB-27 surface, secreted EPS, and reduced cell size (Figure 1A) in contrast to cells cultured in the LB medium, which is rich in nutrients (Figure 1B).

**Figure 1:**
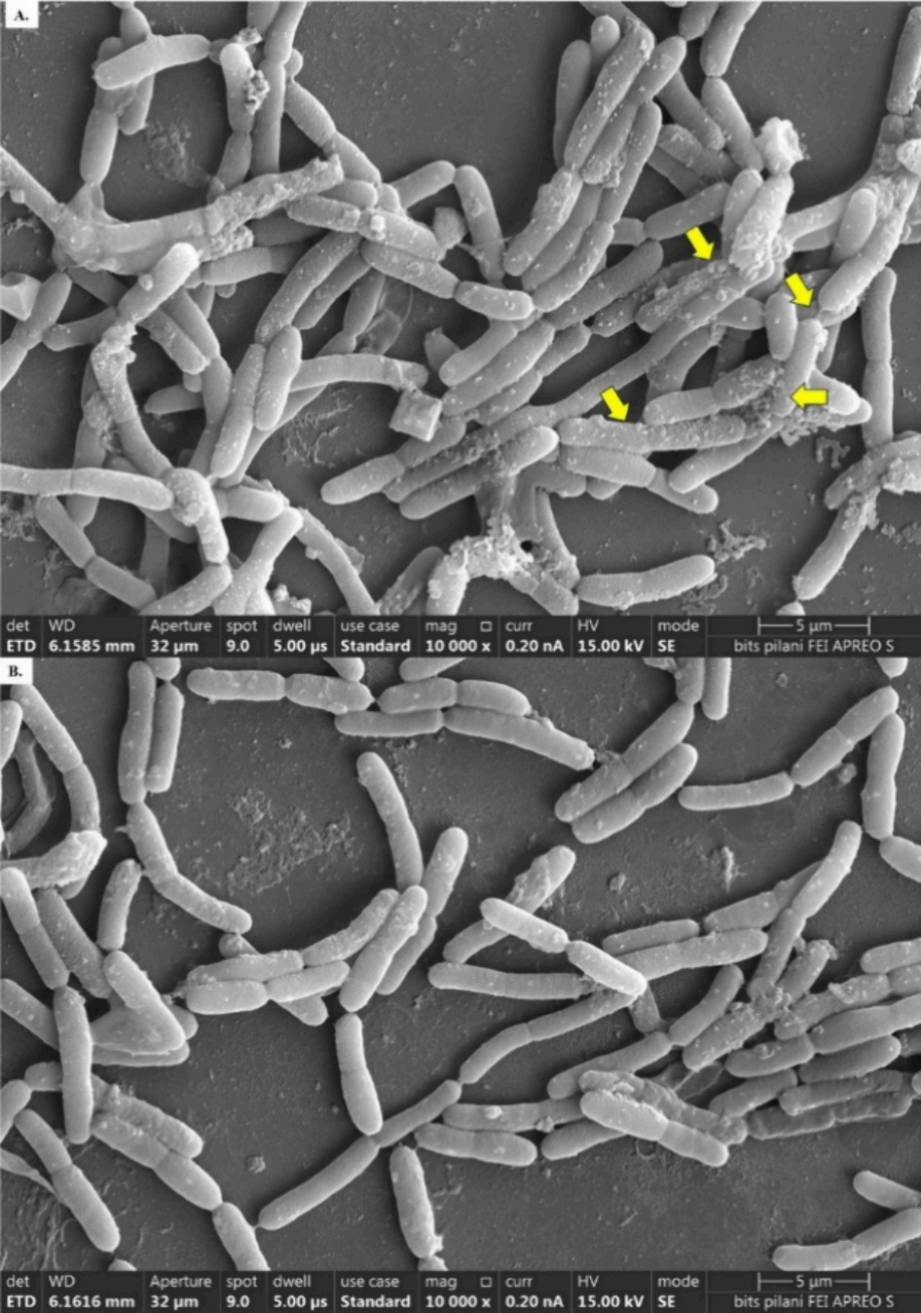
FESEM analysis (A) MAPB-27 grown in biphenyl-treated and (B) control, 10,000× arrow indicates EPS secretion and outer membrane vesicles.

### Chemotaxis Assays toward the Metabolites Produced during Biphenyl Biodegradation

The swarm plate assay and drop assay were used to examine the chemotactic behavior of strain MAPB-27 toward biphenyl/PCB intermediates. Chemotaxis facilitates the bacteria’s interaction with and utilization of the contaminant. Accordingly, this chemotaxis characteristic is crucial to bioremediation^25^ Figure 2B,C displays the successful outcomes of the swarm plate and drop experiments in the form of growth rings following 12–16 h of bacterial incubation. The chemoattractant is positioned in the middle of the semisolid agar plate for the drop experiments, creating a gradient in concentration. MAPB-27 bacterial growth ring was found around the benzoate and catechol placed in the middle of the plates, while diffused growth was observed in biphenyl and DHB.

**Figure 2:**
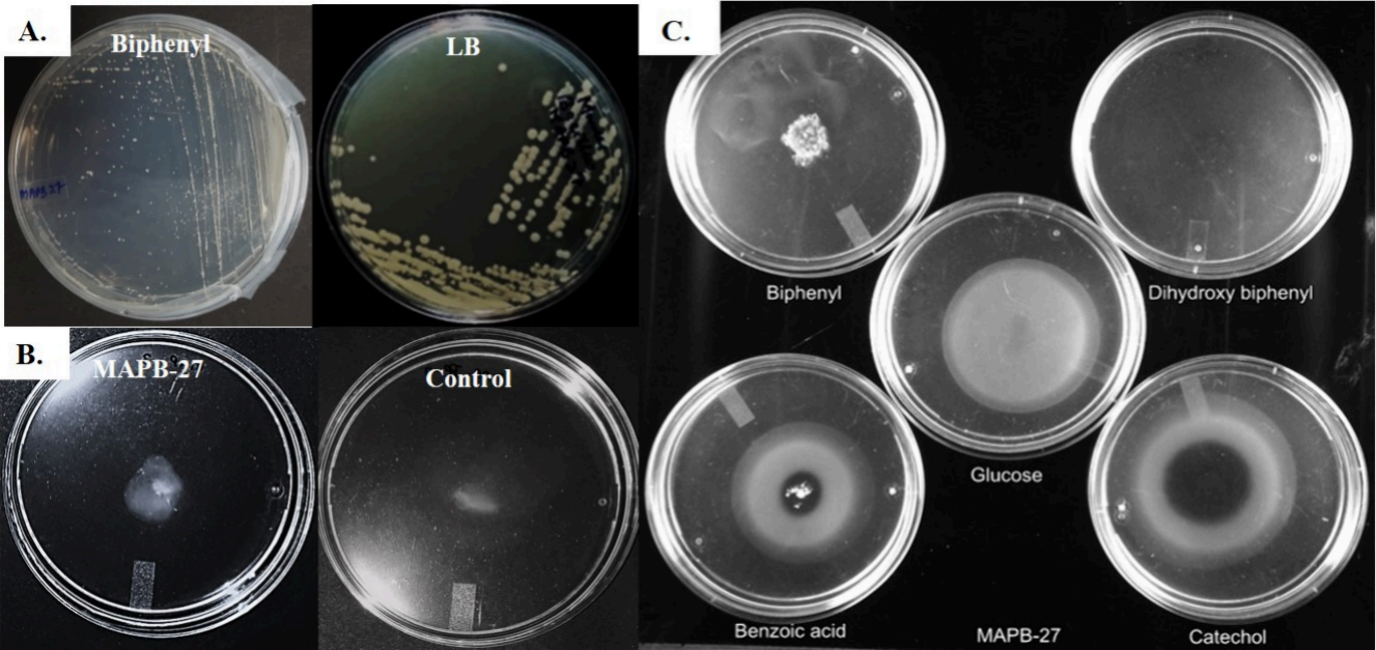
(A) Growth of MAPB-27 in the biphenyl and LB plates. (B) Chemotaxis of the MAPB-27 by Swarm assay. (C) Chemotaxis of the MAPB-27 by drop assay in the presence of intermediates of upper (biphenyl, dihydroxybiphenyl) and lower (benzoic acid, catechol) biphenyl degrading pathway.

These findings showed that the bacterial isolates moved positively toward all substrates, although their growth varied toward the center. According to these findings, strain MAPB-27 is chemotactic toward the four PCB intermediates that were investigated, namely the degradation intermediates of the lower (benzoate and catechol) and upper (biphenyl, dihydroxy biphenyl) biphenyl degradation pathways (Figure 2).

### PCB Congeners’ Biodegradation Potential of MAPB-27

Our previous study provided evidence for the ability of *P. megaterium* MAPB-27 strains to degrade biphenyl and PCB-77.^15^ Therefore, it is essential to evaluate whether the strain MAPB-27 has the potential to degrade tri-, tetra-, penta-, and hexa-chlorinated biphenyls. Isolate *P. megaterium* MAPB-27 degraded 92.5, 62.9, 3.7, and 2.4% trichlorobiphenyl, tetrachlorobiphenyl, pentachlorobiphenyl, and hexachlorobiphenyl, respectively (Figure 3).

**Figure 3:**
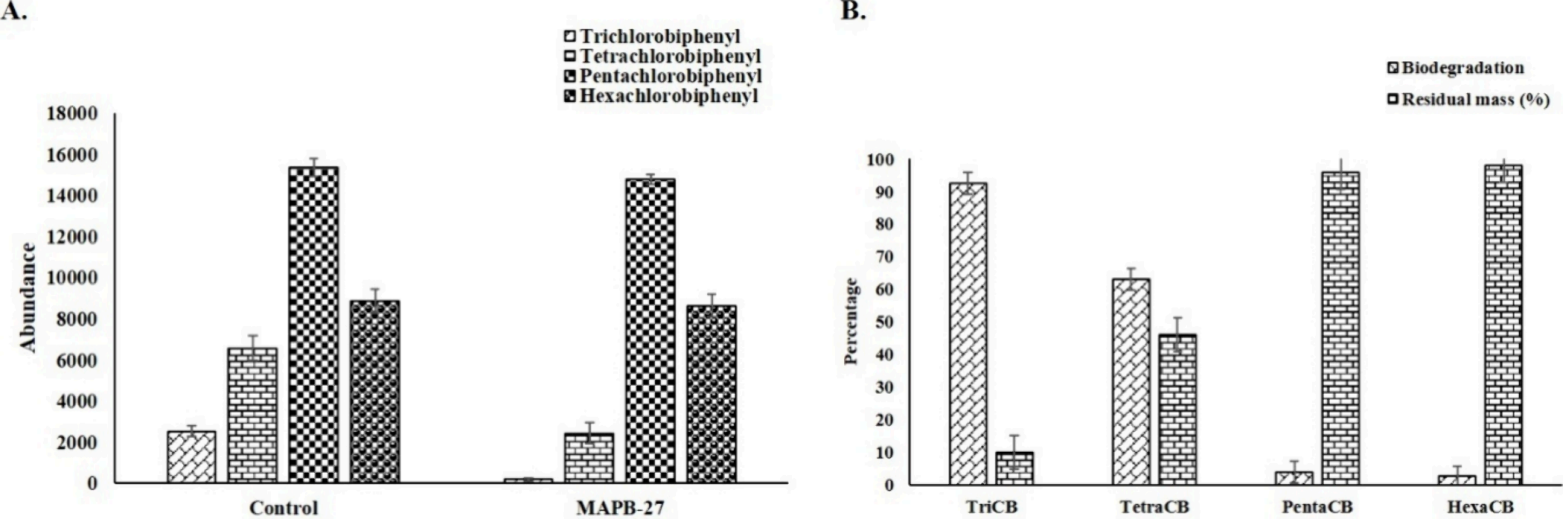
GC-MS/MS analysis of PCB congener mix degradation by the MAPB-27 treatment. The number on the *X*-axis refers to the treatment and control groups. (A) *Y*-axis refers to the abundance of PCB congeners. (B) *Y*-axis refers to the percent degradation and residual mass (%) of PCB congener supplemented to MM by MAPB-27. PCB congeners consist of tri-, tetra-, penta-, and hexa-chlorinated biphenyls.

### Metabolomics Analysis of MAPB-27 Grown in Biphenyl

#### Metabolites for Biphenyl Degradation

Microbial biodegradation integrated with metabolomics is a strategic approach to understanding the breakdown of persistent organic compounds and metabolic pathways. To the best of our knowledge, metabolomic studies of biphenyl-degrading *P. megaterium* remain unknown. To investigate the function of metabolites and pinpoint the metabolic processes involved in stress tolerance brought on by biphenyl, we extracted metabolites and performed GC-MS/MS untargeted metabolomics of *P. megaterium* MAPB-27 grown under control (glucose, 0.2%) and biphenyl-supplemented MM (200 mg L^–1^). GC-MS/MS analysis was conducted to primarily highlight the metabolomics of MAPB-27 upon exposure to biphenyl with 0.2% glucose as a control. As the growth of MAPB-27 in 200 mg/L was equivalent (based on O.D.) to that of 0.2% glucose, the given concentrations were comparable. The metabolites identified were 4-dihydro-2-oxo-valerate, benzoic acid, and 2,3-dihydroxybenzoic acid during the biphenyl degradation MAPB-27 at *R*_t_ 16.5, 17.3, and 25.4 min with significant log_2_(FC) 0.37, 1.06, and 0.89, respectively (Table 1). Trimethylsilyl (TMS) -derived metabolites that were produced in biphenyl and linked to the benzoate breakdown pathway were identified by GC-MS/MS (Figure 4).

**Figure 4:**
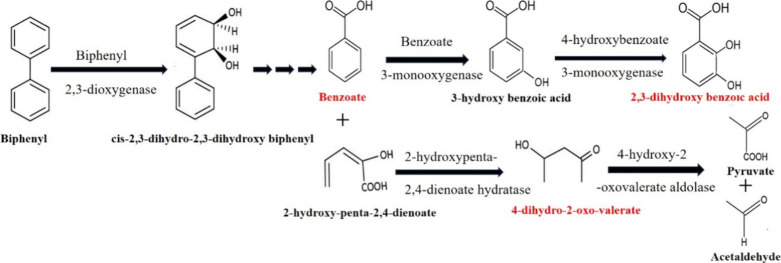
Identified metabolites produced during the degradation process by *P. megaterium* MAPB-27 confirm the biphenyl degradation via 2,3-dioxygenase cleavage. Metabolites in red are identified compounds in the extract of MAPB-27.

**Table 1:** List of Identified Metabolites of *Priestia megaterium* MAPB-27 Produced in the Biphenyl-Stressed and Control Conditions, as Identified by GC-MS/MS Analysis

	metabolites				
1	methoxyamine	36	3-tetradecene, (Z)-	71	palmitic acid
2	acetamide	37	E-11,13-tetradecadien-1-ol	72	4-hydroxyphenyllactic acid
3	2-methyl-4-pentenoiate	38	formic acid	73	D-sorbitol
4	crotonic acid	39	butanedioic acid	74	1-pentadecanol
5	1,4-butanediol	40	benzeneacetic acid	75	heptadecanoic acid
6	boric acid	41	2-octenoic acid	76	N-acetyltyrosine
7	propanedioic acid	42	4-aminobutanoic acid	77	15-tetracosenoic acid, (Z)-
8	ethylene glycol	43	azelaic acid	78	heptadecanoic acid
9	acetic acid	44	3-hexadecene, (Z)-	79	2-hexyldodecanol
10	tiglic acid	45	nonanoic acid	80	stearic acid
11	3-pyridinol	46	tromethamine	81	9-octadecenoic acid, (E)-
12	phenol	47	9-tetradecenoic acid, (E)-	82	hexadecanoic acid
13	D(−)-lactic acid	48	1,2-propanediol-1-phosphate	83	o-coumaric acid
14	acetophenone	49	D-alloisoleucine, N-acetyl-	84	1-tetradecanol
15	hexanoic acid	50	2,5-cyclohexadiene1,4-dione	85	9-hexadecenoic acid, (Z)-
16	glycolic acid	51	9-decenoic acid	86	8-phenyloctanoic acid
17	2,3-butanediol	52	3,5-di*tert*-butyl-4-hppa^b^	87	D- (+)-galactose
18	acetoin	53	2,6-bis(*tert*-butyl) phenol	88	15-tetracosenoic acid, (Z)-
19	halostachine	54	3-phenyllactic acid	89	dehydroabietic acid
20	oxalic acid	55	E-14-hexadecenal	90	chlorophacinone
21	3-hydroxypropionoic acid	56	benzophenone	91	eicosanoic acid
22	benzyl alcohol	57	dodecanoic acid	92	citronellic acid
23	4-pyridinol	58	tridecanoic acid	93	11-eicosenoic acid, (E)-
24	2-hydroxy-2-methylBA^a^	59	phthalic acid	94	n-tetracosanol-1
25	(R)-3-hydroxyBA^a^	60	2,3-dihydroxybenzoic acid	95	13-docosenoic acid, (Z)-
26	heptanoic acid	61	dibutyl phthalate	96	abietic acid
27	2-ketobutyric acid	62	itaconic acid	97	1-monooleoylglycerol
28	l-norvaline	63	n-pentadecanol	98	cyclohexadecane
29	dodecane	64	Z-5-nonadecene	99	dihydrophytol
30	4-decanol	65	isopropyl myristate	100	dodecanoic acid
31	1-dodecene	66	heneicosane	101	8-heptadecanol
32	4-hydroxy-2-oxo-valerate	67	citric acid	102	behenic acid
33	4-hydroxybutanoic acid	68	dodecanoic acid	103	1,2-dipalmitin
34	3-hydroxyisovaleric acid	69	myristic acid	104	beta-sitosterol
35	benzoic acid	70	pentadecanoic acid	105	stigmasta-3,5-diene

a BA represent butyric acid.

b hppa represents hydroxyphenylpropionic acid.

### PCR Amplification of ARHD

The primary enzyme that controls the substrate specificity of PCB/biphenyl breakdown is ARHD. The expression of this enzyme in MAPB-27 was amplified by using PCR. The presence of the dioxygenase gene in MAPB-27 was verified by sequencing and BLASTn analysis of the 202 bp amplified products. The culture was treated with 100, 200, and 300 mg L^–1^ of biphenyl for 72 and 120 h, respectively, to determine the relative expression level of the ARHD gene. At 72 h, MAPB-27’s expression of ARHD increases as the biphenyl concentration increases (Figure 5). On increasing biphenyl concentration (100, 200, and 300 mg L^–1^), the ARHD gene showed a significant (*p* < 0.001) up-regulation up to 5.5-fold in MAPB-27 after a 72 h incubation period. However, the increase in biphenyl concentrations to 100, 200, and 300 mg L^–1^ upregulated the ARHD gene expression to 9.6, 53.8, and 62.6-fold in MAPB-27 after 120 h of the incubation period (Figure 5). This study has shown significant expression activity of the tested genes at different biphenyl concentrations and times.

**Figure 5:**
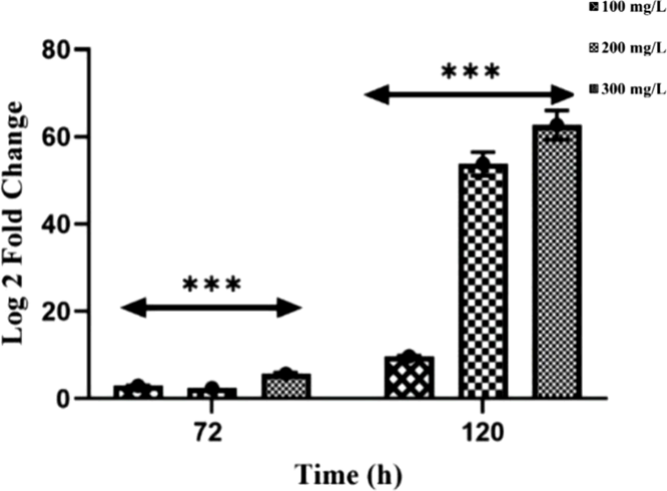
Biphenyl-induced Fold change of expression in ARDH abundance over 16S rRNA by MAPB-27 at different concentrations (100–300 mg L^–1^) supplemented in MM and incubated for 72 and 120 h. An increase in the biphenyl concentration from 100 to 300 mg L^–1^ highly upregulated the ARHD gene expression in MAPB-27. ARHD gene expression was upregulated to 5.5-fold and 62.6-fold at 300 mg L^–1^ for 72 and 120 h, respectively, by MAPB-27.

### Metabolomic Changes in MAPB-27 to Biphenyl Exposure

Metabolomics helps in understanding the cellular responses to combat organic pollutant stimulation. Exposure to organic contaminants leads to alterations in the cellular metabolite profiles. As a result, additional analysis was done on the MAPB-27 extract to look into how biphenyl affected the metabolic processes. The growth of MAPB-27 on biphenyl-supplemented MM and control resulted in the identification of 105 different metabolites (Table 1). It was found that exposure to biphenyl-supplemented MM directly impacted MAPB-27 metabolic activities. The mass spectral NIST14 library used GC-MS/MS analysis to identify the various organic acids, fatty acids, sugars, and amino acids, including glycolic, lactic, oxalic, 1,4-butanediol, palmitic acid, and stearic acid metabolites.

When compared to the control, the metabolites generated by the MABP-27 cultivated on MM supplemented with 200 mg L^–1^ biphenyl after 72 h showed a substantial fold change (Table 2). *P. megaterium* MAPB-27 intracellular metabolites were found to be significantly changed, and the fold change indicated that biphenyl stress raised MAPB-27 metabolite levels. TCA metabolites identified in the biphenyl-supplemented MM extract were citrate and isocitrate, with fold changes of 0.34 and 0.80, respectively. Metabolites such as acetate, glycine, succinate, citrate, glycolate, oxalate, and ethylene glycol identified in the extract of MAPB-27 are well-known metabolites formed during the glyoxylate pathway. Glyoxylate shunting was found to be the potential carbon metabolic strategy adopted by the biphenyl-grown MAPB-27 cell. In addition, 3-hydroxybutyrate and 2-hydroxy-2-methylbutyrate metabolites were found to be present in the extract of MAPB-27 treated with biphenyl. Amino acid metabolites such as citrate and D-Alloisoleucine exhibited significant fold change log_2_(FC) of 0.16 and 1.02, respectively, in the MAPB-27 extract. Metabolites involved in amino acid biosynthesis, such as 4-hydroxybutanoate, 4-aminobutanoic acid, N-acetyl tyrosine, and Norvaline, were identified in the MAPB-27 extract supplemented with biphenyl rather than in the control. Furthermore, 3-hydroxyisovaleric acid, a byproduct of the metabolism of leucine, was found to be present in the extract.

**Table 2:** Identified Metabolites of *Priestia megaterium* MAPB-27 with Substantial Fold Change in the Biphenyl Stressed State

sr. no.	metabolite	log_2_(FC)	sr. no.	metabolite	log_2_(FC)
**1**	methoxyamine	–1.02	**19**	D-galactose	0.32
**2**	crotonic acid	0.68	**20**	dodecanoic acid	1.02
**3**	tiglic acid	0.08	**21**	phthalic acid	0.45
**4**	3-pyridinol	–0.39	**22**	2,3-dihydroxybenzoic acid	0.89
**5**	phenol	1.57	**23**	itaconic acid	0.80
**6**	D-(−)-lactic acid	1.15	**24**	rhamnose	0.63
**7**	acetophenone	–0.87	**25**	Z-5-nonadecene	–1.02
**8**	hexanoic acid	2.02	**26**	palmitic acid	1.33
**9**	glycolic acid	0.75	**27**	myristic acid	0.59
**10**	oxalic acid	1.02	**28**	pentadecanoic acid	1.33
**11**	2-hydroxy-2-methylbutyric acid	0.95	**29**	heptadecanoic acid	0.64
**12**	2-ketobutyric acid	–1.02	**30**	15-tetracosenoic acid, (Z)-	–0.25
**13**	butanedioic acid	1.02	**31**	heptadecanoic acid	1.31
**14**	citrate	0.16	**32**	stearic acid	2.36
**15**	benzeneacetic acid	–1.02	**33**	9-octadecenoic acid, (E)-	–0.41
**16**	tromethamine	–1.02	**34**	trans-13-octadecenoic acid	0.62
**17**	D-alloisoleucine, N-acetyl-	1.02	**35**	eicosanoic acid	0.89
**18**	2,6-bis(*tert*-butyl) phenol	–1.02	**36**	behenic acid	0.01

Further, alkylated salicylic acid (6-[12­(Z)-nonadecenyl] salicylic acid), a derivative of salicylic acid, and certain sugar moieties such as D-galactose, D-sorbitol, and rhamnose were also observed in the extract of MAPB-27. D-galactose is a lactose hydrolysis product that is believed to be an exopolysaccharide (EPS) component of the *Bacillus* biofilm matrix.^26^ According to FESEM analysis, they are seen to concentrate extracellularly on the cell surface.

To maintain the energy level, bacteria produce many fermentation byproducts. 2,3-butanediol, acetoin, malonic (propanedioic acid), lactic, and butanedioic acid (succinic acid) were identified in the MAPB-27 extract grown in biphenyl-supplemented MM. The lactic acid and butanedioic acid (succinic acid) showed significant log_2_(FC) of 1.1 and 1.2, respectively (Table 2). In addition, 3-hydroxypropionoic acid (3-HP), known as hydracrylic acid, was found to be present in the MAPB-27 extract supplemented with biphenyl. Acetic acid bacteria are reported to convert 1,3-propanediol (1,3-PDO) into 3-HP.^27^ 3-HP is one of the most promising biobased building blocks with a wide range of direct industrial applications.^28^

#### Fatty Acid Accumulation in MAPB-27 Grown in Biphenyl-Supplemented MM

The cell membrane of bacteria is altered as a survival adaptation mechanism. When biphenyl was present, there was a comparatively greater amount of saturated fatty acids than in the control (Figure 6). The TMS derivatives of stearic acid, 11,14-eicosanoic acid, palmitic acid, 5-dodecenoic acid, trans 13-octadecanoic acid, heptadecanoic acid, pentadecanoic acid, and myristic acid indicated a higher fatty acid percentage with log_2_(FC) of 2.36, 0.89, 1.33, 1.02, 0.62, 0.64, 1.33, and 0.59, respectively, in the biphenyl-induced MAPB-27.

**Figure 6:**
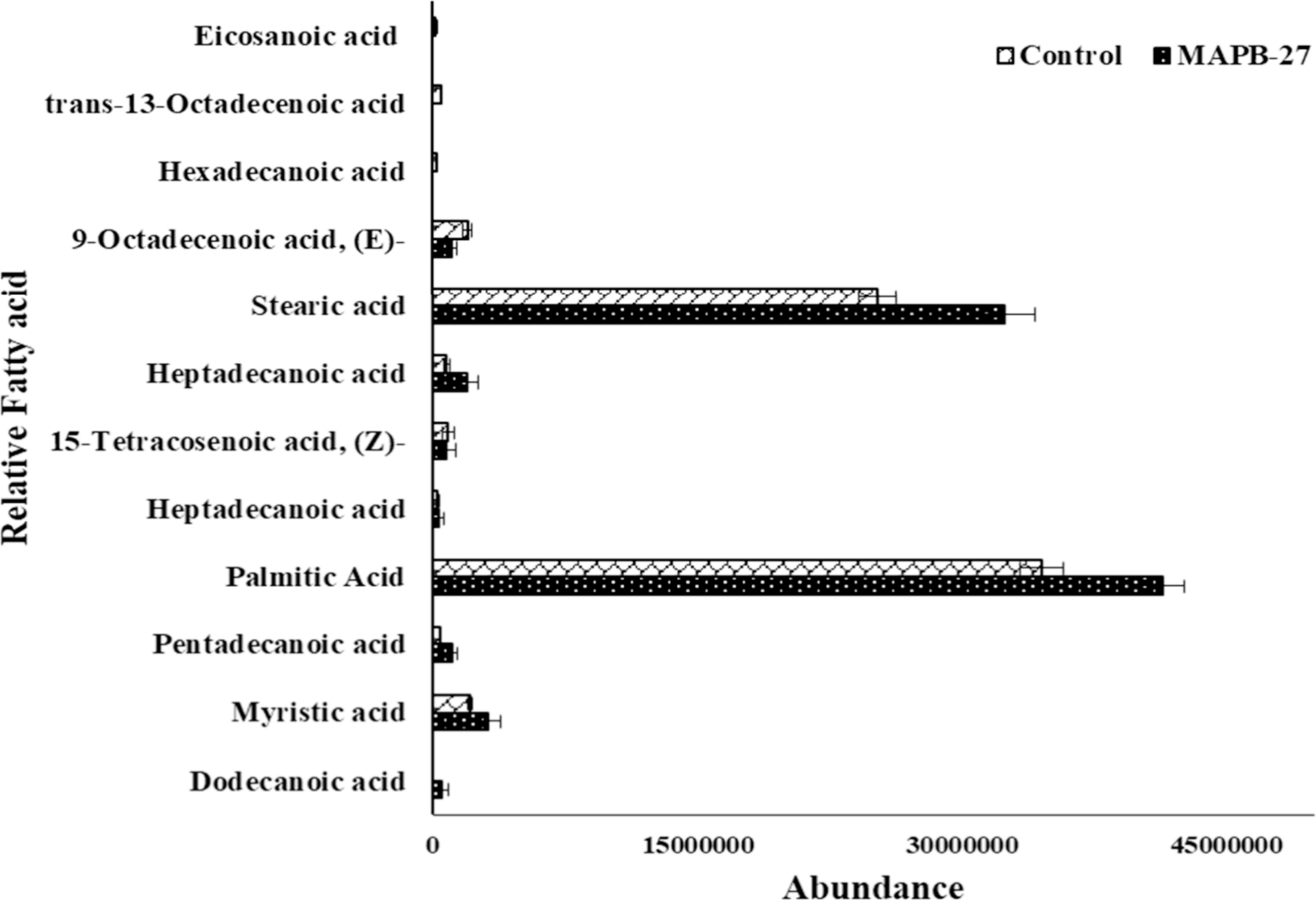
Comparative abundance of the fatty acid profile of MAPB-27 in MM with biphenyl supplementation and control.

#### Accumulation of Antifungal and Antimicrobial Compounds by MAPB-27

Numerous bioactive substances, including those with antibacterial and antifungal properties, are known to be bacterial metabolites. Metabolites, including behenic acid, palmitic acid, 4-hydroxyphenyllactic acid (HPLA), β-sitosterol, and 3-phenyllactic acid (PLA), were identified in the ethyl acetate extract of *P. megaterium* MAPB-27.

#### Metabolite Set Enrichment Analysis

The KEGG database served as the basis for enrichment analysis of the variation in metabolic pathways. Figure 7 displays a bubble diagram representing differential metabolites in which the enrichment factor is known as a Rich Factor. Fatty acid biosynthesis and glyoxylate and dicarboxylate metabolism were demonstrated to be active metabolism in MAPB-27 by enrichment metabolite analysis. As depicted in Figure 7, two main enriched metabolic pathways are glyoxylate and dicarboxylate, and fatty acid metabolism in biphenyl-supplemented MM. Higher fatty acids enter the glyoxylate cycle when they are oxidized to acetyl-CoA without producing pyruvate.

**Figure 7:**
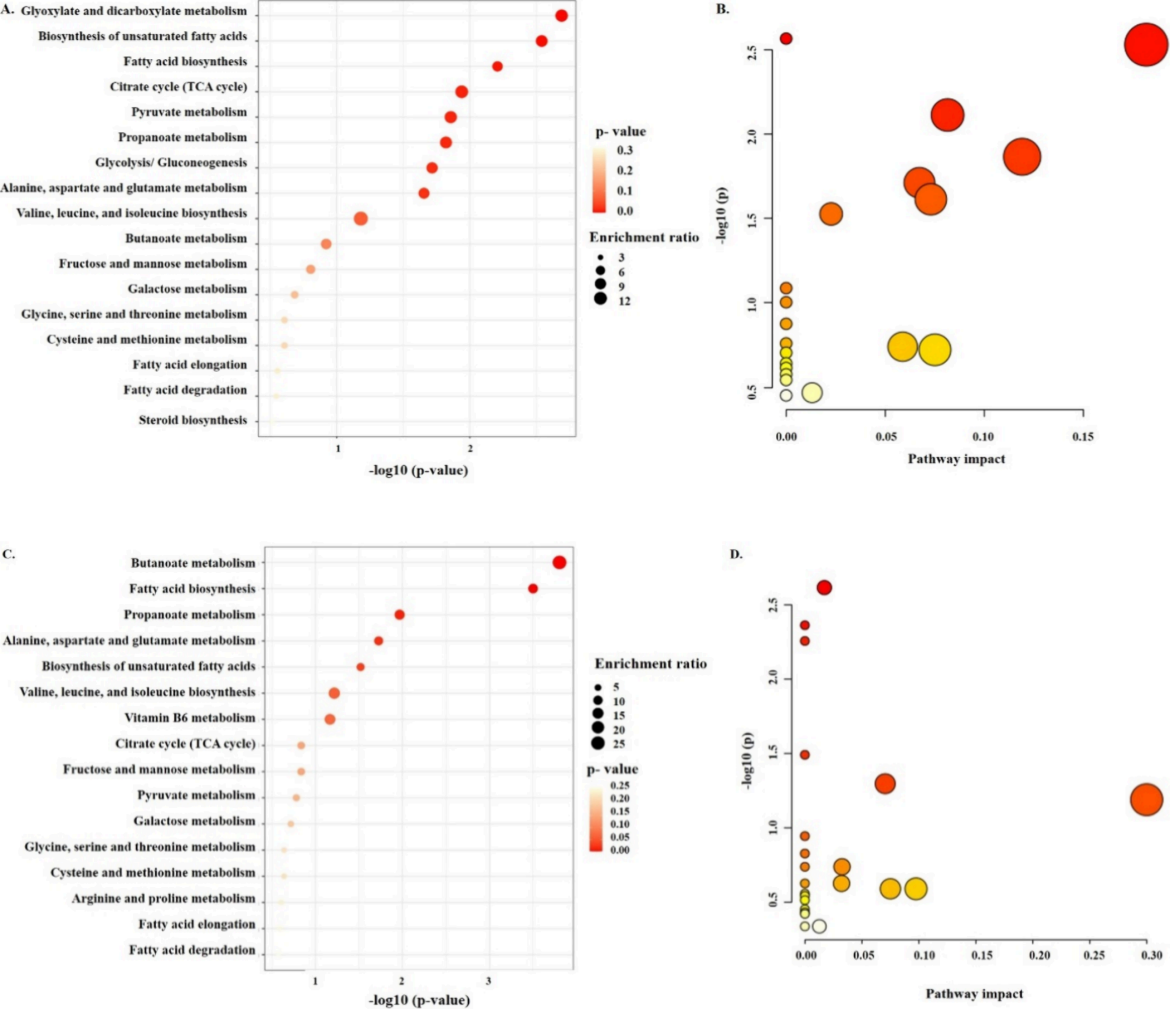
Metabolite Set Enrichment Analysis: biologically significant and enriched patterns in the metabolomic data. MetaboAnalyst 5.0 software was used to analyze metabolomic data. The study’s input for identifying the metabolic pathway in MAPB-27 was the metabolites found by the GC-MS/MS analysis. (A,B) under biphenyl-induced stress conditions and (C,D) control. Hits = observed hits; expected = expected hits is the formula used to calculate the enrichment ratio. The most active metabolisms in MAPB-27 in biphenyl-supplemented MM were found to be glyoxylate and dicarboxylate. The number of differential metabolites within the entry is represented by the increased size of the bubble. The *P*-value is represented by the color of the bubble. The significance of the enrichment increases with a smaller *P*-value.

## Discussion

The most researched organisms for PCB bioremediation are bacteria because they are ubiquitous and may flourish in a PCB-contaminated environment. One of the best alternative methods for eliminating PCBs from soil is bioremediation. Isolating potential bacteria that break down PCBs appears to be a viable way to improve PCB remediation. This study intends to describe its PCB biodegradation ability and metabolic reaction, as well as offer novel applications for this strain, in response to the paucity of research on its broader capabilities and traits. Our lab previously isolated and characterized MAPB27 (accession number MK512377.1) from PCB-contaminated nearby soil of Bhilai Steel plant, Chhattisgarh, and reported its PCB-degrading property.^15^ Given its function in degradation and the fact that it was isolated from a PCB-contaminated region, we have theorized that this PCB-degrading capacity may possess special stress adaptation capabilities.

We first evaluated the growth, morphology, and chemotaxis of MAPB-27 under biphenyl exposure to validate our hypothesis. MM enriched with biphenyl and nutrient-rich LB media were used to study MAPB-27 growth, because biphenyl can inhibit bacterial growth. Figure 1 makes it clear that MAPB-27 grew and survived on both media plates. The bacterium *P. megaterium* is large and rod-shaped. The bacterial isolate showed a typical bacilli shape in the LB medium. However, slower growth was observed than in its nonstressed counterparts. Bacterial cells may have focused their energy flow on triggering defense systems under stress, which could have impacted their growth. On exposure to biphenyl stress, we saw the development of vesicle-like structures. According to one such study, Bacillus secretes EPS on its cell surface when exposed to organic contaminants like phenanthrene.^29^ Bacterial membrane vesicles (BMVs) have been reported to arise as an adaptation mechanism to environmental stress.^30,31^ When *Burkholderia xenovorans* LB400 biphenyl-grown cells were examined using scanning electron microscopy (SEM), physiological alterations such as cell shape, size decrease, and membrane separation were observed. Additionally, the formation of vesicles in a bacterial cell due to exposure to chemicals, such as toluene and biphenyl/PCBs, is often a sign of stressful conditions.^32^ These vesicles may have a specific activity, such as forming biofilms or acquiring nutrients, and they are crucial in host-microbe interactions, which allow them to withstand adverse environmental circumstances.

Benzoate and CBAs are among the intermediates produced as a result of biphenyl/PCB degradation.^33^ Our results indicate that MAPB-27 demonstrated chemotaxis toward intermediates of benzoate, catechol, dihydroxy biphenyl, and biphenyl. Bacterial proliferation around the chemoattractant is an indication of chemotaxis. The chemotaxis of *Pseudomonas* species^34,35^ and *Rhodococcus* species^36^ toward chemicals has been reported in the past. Researchers have examined the chemotactic response of PCB-degrading bacteria to benzoate, biphenyl,^37^ PCBs,^38^ and CBA.^39^ Additionally, the biodegradation of PCB congeners tri-, tetra-, penta-, and hexa-chlorinated validates MAPB-27’s capacity for biodegradation. Our study shows that after 72 and 120 h, 100 mg L^–1^ biphenyl induction and expression of the ARHD gene in the MAPB-27. The expression of ARHD in MAPB-27 is higher at 72 h when the concentration is higher (i.e., 300 mg L^–1^). The increase in MAPB-27 may be due to the stress that biphenyl produces to initiate the biphenyl breakdown pathway by boosting the expression of ARHD. Biphenyl dioxygenases are type IV multicomponent enzymes of ARHDs, initiating the aerobic breakdown of biphenyl, converting it into cis-2,3-dihydro-2,3-dihydroxy biphenyl (dihydrodiol compound).^40^ According to reports, this enzyme opens the aromatic ring by adding two hydroxyl groups.^41,42^ These results imply that the physical ambient parameters, including concentration and incubation time as well as the accessible inducer biphenyl, affect the expression of ARHD in the selected PCB-degrading bacteria. Master and Mohn^43^ also observed notable differences in the effects of physical and chemical ambient variables on the genetic regulation of PCB breakdown in different bacteria. Some publications claim that biphenyl causes *Pseudomonas* sp. KKS102 to express more ARHD.^44^

We examined the metabolic reactions of MAPB-27 exposed to biphenyl to gain a better understanding of the stress adaptation process. We used GC-MS/MS-based untargeted metabolomics to ascertain the changes in primary and secondary metabolites under biphenyl. A deeper understanding of the biphenyl breakdown was made possible by the deregulation of these intracellular metabolites, which showed how central carbon and nitrogen metabolism responded to biphenyl stress. When exposed to organic pollutants, such as PAH and PCBs, the bacterial cell has a tendency to save energy. This need has been linked to a variety of stress response mechanisms, including metabolic alterations. Deregulated metabolites were separated into a number of types according to their roles in various metabolic pathways, biochemical structures, and activities. The presence of 4-dihydro-2-oxo-valerate, benzoate, and 2,3-hydroxybenzoic acid in the biphenyl-supplemented MAPB-27 extract suggests that the bacterial isolate degrades biphenyl. The discovery of identified metabolites affects many metabolic processes, such as the tricarboxylic acid cycle (TCA cycle), glyoxylate shunt, pyruvate metabolism, amino acid biosynthesis, propionate metabolism, and fatty acid metabolism. According to several sources, the bacteria build up oxalates when they are under stress. The metabolic interaction of the glyoxylate cycle and TCA produces oxalate. Oxalate has been shown to chelate metals, which may lessen oxidative stress.^45^ Some bacteria have an adaptability process called the glyoxylate cycle that turns fatty acids into carbohydrates when they need energy from organic sources. Oxidative stress caused by organic pollutants such as biphenyl/PCB alters the structure of cells, alters the fatty acid composition, and alters different proteins. Additionally, it was demonstrated that the extract included metabolites that are involved in the glyoxylate pathway. Bacteria such as *P. megaterium* have been shown to use fatty acids and acetate as their exclusive carbon sources through the glyoxylate pathway. 3-hydroxybutyrate (3-HB) and 2-hydroxy-2-methylbutyrate metabolites were found to be present in the extract of MAPB-27 treated with biphenyl. 3-HB is primarily a monomeric unit of poly-3-hydroxybutyrate (PHB), a reserve material that serves as a significant source of energy and carbon in microorganisms. It is produced by bacteria of the genera *Bacillus*, *Pseudomonas*, *Rhizobium,* and *Ralstonia*.^46,47^ Since the breakdown of biphenyl requires a lot of energy and survival under stress is an energy-demanding activity, the activation of the glycolytic and TCA cycles and production of reserve materials may be implicated in meeting these demands. Increased levels of glycolysis and TCA cycle metabolites may give the bacterial cell energy in the form of ATP when it is under biphenyl stress.

It has also been demonstrated that bacterial cells under stress can accumulate amino acids.^48^ After stress exposure, several metabolites that are involved in the production of amino acids, including D-alloisoleucine, 4-hydroxybutanoate, 4-aminobutanoic acid, N-acetyl, tyrosine, and norvaline, were accumulated. According to reports, norvaline is synthesized and accumulated more quickly in cells with increased pyruvate accumulation.^49^ Additionally, MAPB-27 exposed to biphenyl had an excess of volatile organic compounds (VOCs). The screw cap vials may eventually suffer low oxygen levels or an anaerobic state since the selected microorganisms are facultative anaerobes.^50^ Acetoin is recognized to be an intermediary step in the fermentation of 2,3-butanediol and has been found in *P. megaterium* MAPB-27. It has been reported that Bacillus strains use substrates such as glucose, starch, galactose, cellulose, and glycerol to produce acetoin in fermentative conditions.^51,52^ According to reports, *Acetobacterium woodii* catabolizes 2,3-butanediol via a route that includes acetoin, acetaldehyde, and acetyl-CoA. In order to help bacteria use 2,3-butanediol as a carbon source and aid in the breakdown of contaminants, this method combines with the Wood-Ljungdahl pathway.^53^ The cells exposed to biphenyl showed a greater buildup of salicylic acid. According to reports, salicylic acid is a secondary metabolite produced by most bacteria, including *Pseudomonas*, *Achromobacter*, and *Mycobacterium*.^54–56^ In order to counteract biotic and abiotic stress processes, *Bacillus* species are also known to manufacture SA, which is then integrated into salicylate-based siderophores. Its potential to cause PCB mineralization is highlighted by reports that SA induces PCB breakdown.^57^ With a notable up-regulation of the relative expression of the functional gene bphA1, SA verified the induction of PCB degradation in *Pseudomonas fluorescens* P2W.^57^

Additionally, metabolomics data showed that MAPB-27 under biphenyl stress had higher concentrations of sugar compounds, such as sorbitol, D-galactose, D-lactose, and rhamnose. According to reports, a greater quantity of D-galactose, glucose, and rhamnose is needed for the synthesis of EPS and the biosurfactant in order to support cell viability and biphenyl breakdown. EPS production is induced by different abiotic stresses such as heat stress, salt tolerance, drought, and nutrient deficit.^58,59^ Consequently, increased sugar content may improve cell hydrophobicity, protect cells against biphenyl stress, and aid in the synthesis of EPS and biosurfactants. According to reports, *P. aeruginosa* B1, *P. fluorescens* B5, *P. stutzeri* B11, *P. putida* B15, and *P. megaterium* produce a lot of EPS when cultivated in MM supplemented with different organic pollutants as gasoline, 2,4-D, benzene, BTX, and biphenyl.^60,15^ Further, some reports highlight the production of biosurfactants with increased cell hydrophobicity, which influences the rate of biodegradation of persistent PAH chemicals, such as pyrene and fluorene,^61,62^ and chlorinated pollutants such as biphenyl/PCBs.^63^

Cell adaptation, including the accessibility of the biphenyl to the bacterial cell for breakdown, may be the cause of the comparatively high amount of saturated fatty acids seen in the MAPB-27 extract. According to earlier research, higher concentrations of completely saturated fatty acids, such as stearic and palmitic acids, cause the cell membrane to stiffen, which lowers the permeability of the membrane to tiny molecules^64^ under stress. Similar metabolomic findings were observed with *Methylorubrum* sp. ZY-1 cell was exposed to PCB-118 (2,3′,4,4′,5-pentachlorobiphenyl), resulting in up-regulation of palmitic acid and dodecanoic acid levels. ZY-1 strain on exposure to PCB-118 regulated fatty acid composition as an adaptive mechanism to combat stress.^37^ Thus, the buildup of fatty acids in the extracts of the bacterial isolate MAPB-27 suggested a metabolic approach to counteract nutritional stress brought on by biphenyl.

These metabolic responses and alterations in cellular metabolism might be related to active fatty acid biosynthesis and glyoxylate and dicarboxylate metabolism in MAPB-27. The glyoxylate pathway is linked to the metabolism and production of fatty acids. According to a prior study, PCB buildup causes bacterial membranes to become more saturated with fatty acids.^65^ However, butanoate and propanoate were found to be prominent metabolites in the control as compared with biphenyl-exposed cells. Therefore, the discovery highlights how MAPB-27, when exposed to biphenyl, combats stress by converting the biphenyl into metabolites and triggering the glyoxylate pathway, which provides cellular precursors from a single readily available carbon source (Figure 8).

**Figure 8:**
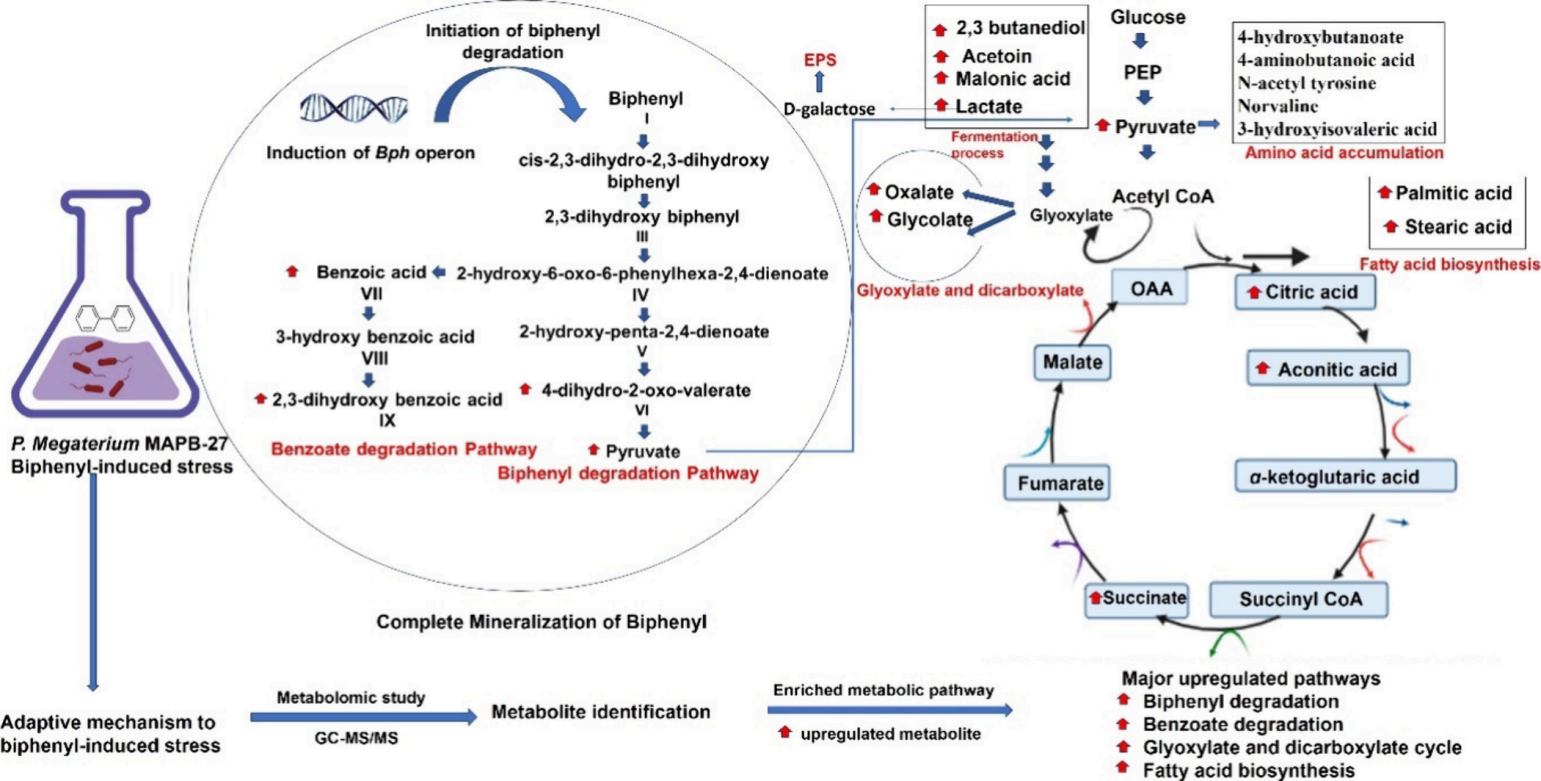
Overview of the MAPB-27’s metabolomic profile after exposure to biphenyl. The red arrow indicates a high level of metabolism.

Finally, we also observed an accumulation of antifungal and antibacterial metabolites, such as palmitic acid, behenic acid, β-sitosterol, 3-phenyllactic acid (PLA), and 4-hydroxyphenyllactic acid (HPLA). *P. megaterium* is renowned for producing metabolites with antifungal and antibacterial activities.^66–68^ β-sitosterol, PLA, and HPLA are well-known antibacterial drugs produced by *Bacillus* sp.^69^ and several lactic acid bacteria.^70^ According to reports, PLA and HPLA are metabolic byproducts produced by bacteria when phenylalanine (Phe) and tyrosine (Tyr) are broken down, respectively. These amino acids undergo transamination to produce 2-keto-carboxylic acids, such as HPPA and phenylpyruvic acid. The accumulation of 3-hydroxybutyrate is an adaptation mechanism of *P. megaterium* to combat nutritional stress.^71^ It is reported that acetyl-CoA is directed to the PHB biosynthetic pathway during nutrient deficiency, while its entry into the Krebs cycle is restricted.

## Conclusion

The present study reports chemotaxis, PCB degradation, and metabolic responses of *P. megatarium* MAPB-27 to biphenyl. *P. megatarium* MAPB-27 degrades PCB congeners having 3–4 chlorinated biphenyls with 92.5–62.9% efficiency. Moreover, exposure to PCB congeners affects the size of bacteria, as observed in FESEM. Interestingly, the metabolite profile characteristic of bacteria was different when cultured under biphenyl-supplemented media. Overall, the results of the metabolic enrichment analysis, metabolomic profiling, and cell morphology indicated that MAPB-27 demonstrated an adaptive mechanism to survive under stress conditions caused by biphenyl. The identification of biodegradative metabolites such as 4-dihydro-2-oxo-valerate, benzoic acid, and 2,3-hydroxybenzoic acid highlights the induction of the enzymes linked to biphenyl and benzoic acid degradation pathways. In addition to biodegradation pathways, glyoxylate and fatty acid biosynthesis pathways were also found to be positively regulated in response to biphenyl. Accumulation of metabolites, such as oxalate, acetoin, 2,3-butanediol, salicylic acid, and 3-hydroxybutyrate, indicates an adaptive mechanism to combat stress. Further, compounds like poly 3-hydroxybutyrate (3-hydroxybutyrate), biobased organic acid (3-hydroxypropionoic acid), antibacterial and antifungal (phenyllactic acid, 4-hydroxyphenyllactic acid, and β-sitosterol), produced in response to biphenyl stress, can have a wide range of direct industrial applications. Thus, the present work identifies metabolites involved in biphenyl degradation and other key metabolites required as an adaptive mechanism to nutritional stress in *P. megaterium* MAPB-27. Our results suggest that *P. megaterium* MAPB-27 is equipped with machinery to efficiently degrade PCBs and, hence, can be applied to the environment for the bioremediation of PCBs. It also increases the understanding of the cellular processes that can be further explored for bioremediation and industrial applications.

## Data Availability

Data will be made available from the corresponding author on reasonable request.
